# Spindle-to-oocyte light retardance ratio as a noninvasive biomarker for oocyte quality assessment: a prospective cohort study

**DOI:** 10.3389/fendo.2026.1803476

**Published:** 2026-05-05

**Authors:** Chun-I Lee, Hsiu-Hui Chen, Wei-Che Lo, Chun-Chia Huang, Tsung-Hsien Lee, Pin-Yao Lin, Ming-Jer Chen, Kuang-Han Chao, Maw-Sheng Lee, Chien-Hong Chen

**Affiliations:** 1 Division of Infertility, Lee Women’s Hospital, Taichung, Taiwan; 2 Department of Obstetrics and Gynecology, Chung Shan Medical University Hospital, Taichung, Taiwan; 3 Institute of Medicine, Chung Shan Medical University, Taichung, Taiwan; 4 Department of Obstetrics and Gynecology and Women’s Health, Taichung Veterans General Hospital, Taichung, Taiwan; 5 School of Medicine, National Yang-Ming Chiao Tung University, Taipei, Taiwan; 6 Department of Obstetrics and Gynecology, National Taiwan University Hospital, Taipei, Taiwan

**Keywords:** light retardance ratio, morphokinetics, oocyte, polarized light imaging, usable blastocyst formation

## Abstract

**Background:**

Polarized light (PL) imaging provides a noninvasive strategy to assess oocyte quality and maturity. This prospective study aims to identify PL parameters in metaphase II (MII) oocytes that serve as adjunctive indicators for usable blastocyst formation.

**Methods:**

From August 2024 to August 2025, we studied 581 mature oocytes from women aged ≤ 38 years undergoing autologous *in vitro* fertilization cycles at Lee Women’s Hospital. Before intracytoplasmic sperm injection, we used PL microscopy to measure birefringence signals in the spindle and zona pellucida (ZP). Fertilized embryos were cultured in a time-lapse (TL) system. We then categorized the oocytes into groups based on their birefringence signals to compare their developmental potential.

**Results:**

Through PL imaging, we identified the oocytes with superior developmental competence. Oocytes with a visible MII spindle achieved significantly higher developmental rates—notably for usable blastocyst development—than did those without an MII spindle (46.1% vs. 15.6%). MII oocytes with high spindle and ZP birefringence demonstrated the highest usable blastocyst rate (53.7%) and spindle-to-oocyte light retardance ratio (SOLRR; 0.011 ± 0.003). In multivariate analysis, SOLRR and anti-Müllerian hormone were significant positive and negative predictors of usable blastocyst formation (adjusted odds ratios = 1.148 and 0.939, respectively). SOLRR alone demonstrated moderate predictive power (area under the receiver operating characteristic curve = 0.63). Oocytes with high SOLRR (≥0.01127) consistently outperformed those with low SOLRR (<0.01127), achieving a higher usable blastocyst rate (61.8% vs. 38.0%). In TL analysis, the high SOLRR group demonstrated faster embryonic progression than the low SOLRR group did, indicated by faster blastocyst formation (108.2 vs. 111.2 h) and blastocoel expansion (8.7 vs. 10.2 h); this group also achieved a higher nonreverse cleavage rate (95.4% vs. 86.8%) and superior trophectoderm morphology (58.0% vs. 40.7%).

**Conclusions:**

PL imaging can be used to identify oocytes with superior developmental potential. SOLRR serves as an exploratory but quantifiably objective indicator of oocyte competence, independently predicting oocytes with relatively high usable blastocyst formation rates, rapid embryonic progression, and improved trophectoderm morphology.

## 1 Introduction

Oocyte quality, the primary factor limiting female fertility, is determined by subcellular structure, mitochondrial remodeling, and biochemical integrity. It governs every early developmental stage, from fertilization through embryonic cleavage to blastocyst formation (1–3). Accurately evaluating oocyte quality is crucial to the effectiveness of modern *in vitro* fertilization (IVF); however, standard morphological assessments are subjective and unreliable indicators of developmental potential. This indicates a need for objective, noninvasive techniques for identifying and selecting the highest-quality oocytes (2, 3).

Polarized light (PL) microscopy can facilitate noninvasive, real-time assessment of oocyte quality by enabling visualization of meiotic spindle and zona pellucida (ZP) signals (3). Structures like the meiotic spindle and zona pellucida split incident PL into two orthogonal rays (**Supplementary Figure 1**). The resulting velocity difference between these rays produces a phase shift, quantified as retardance (4). These metrics are strongly correlated with both oocyte organization and chromosomal alignment (5). The presence, morphology, and intensity of the metaphase II (MII) spindle are correlated with efficient fertilization, normal cleavage, blastocyst development, and embryonic ploidy (6–10). Additionally, categorization of ZP birefringence is a vital indicator of an oocyte’s molecular organization: the higher the ZP birefringence of an oocyte is, the better are the resulting developmental competence and pregnancy outcomes (11–13). Moreover, LC-PolScope—a microscopic imaging system integrating PL with computerized processing for data analysis—aids in effectively quantifying optical retardance on the basis of the intrinsic birefringence of the ZP and microtubules within the spindle (4, 14). However, although early insights have been obtained through qualitative evaluation, the predictive power of quantitative PL parameters for blastocyst development (15, 16) and successful pregnancy (17–21) remains unclear. The aforementioned inconsistencies may have resulted from variations in imaging or evaluation protocols, subjective interpretation of oocyte component birefringence, and high variability in quantitative analysis among studies (22, 23). To address these limitations and enhance clinical utility, a comprehensive, reproducible parameter must be established that effectively integrates spindle and oocyte organization data. Therefore, herein, we propose a novel objective oocyte assessment parameter: the spindle-to-oocyte light retardance ratio (SOLRR). Determining the SOLRR can enable quantitative measurement of relative birefringence between the spindle and the oocyte. In this study, we determined whether the SOLRR can be considered a reliable, single indicator of oocyte competence by validating its associations with *in vitro* embryonic development and time-lapse (TL) morphokinetic outcomes.

## 2 Materials and methods

### 2.1 Study design and cohort

This was a prospective cohort study that included clinical data from women undergoing autologous cycles at Lee Women’s Hospital, Taiwan, from August 2024 to August 2025. Our study protocol was approved by the Institutional Review Board of Chung Shan Medical University, Taichung, Taiwan (approval number: CS2–23192). All patients provided signed informed consent before enrollment. We included ≤ 38-year-old women with a body mass index (BMI) of 18.5–30 kg/m^2^ and an anti-Müllerian hormone (AMH) level of > 1.1 ng/mL who agreed to undergo intracytoplasmic sperm injection (ICSI) and TL cultivation. However, we excluded patients who presented with severe oligoasthenoteratozoospermia and those who used cryopreserved oocytes, surgical sperm retrieval, donor oocytes, or donor sperm.

### 2.2 Controlled ovarian stimulation protocol

We used our established progestin-primed ovarian stimulation protocol (24). First, we initiated controlled ovarian stimulation on cycle day 3 by using follitropin alfa (Gonal-f; Merck-Serono, Darmstadt, Germany), human menopausal gonadotropin (Menopur; Ferring, Pymble, Australia), or both. We administered these agents until two dominant follicles measured ≥17 mm. Medroxyprogesterone acetate (Provera; Pfizer, Milan, Italy) was administered concurrently to suppress pituitary function and prevent a premature luteinizing hormone (LH) surge. Oocyte maturation was triggered with recombinant human chorionic gonadotropin (Ovidrel; Merck-Serono), triptorelin (Decapeptyl; Ferring, Kiel, Germany), or both. After approximately 36 h, ovum pick-up was performed.

### 2.3 IVF and embryo culture

Only mature oocytes with a defined polar body 1 (PB1) were selected for ICSI. Sperm quality was evaluated according to the WHO Laboratory Manual (25). Sperm presenting with oligozoospermia, asthenozoospermia, or teratozoospermia were categorized as abnormal. Fertilized embryos were cultured to the blastocyst stage in a TL culture system (EmbryoScope+; Vitrolife, Kungsbacka, Sweden) with sequential media (SAGE Biopharma, NJ, USA) at 37 °C under 5% O_2_, 6% CO_2_, and 90% N_2_. The normal fertilization rate was calculated as the number of two-pronuclei (2PN) zygotes with polar body 2 (PB2) divided by the number of ICSI oocytes. The full-blastocyst rate was calculated as the number of blastocysts that began to push the ZP divided by the number of ICSI oocytes. Consistent with our previous methodology (26, 27), the usable blastocyst rate was defined as the number of day 5 and day 6 blastocysts reaching a diameter of ≥150 μm with considerable expansion (grade 4, 5, or 6), appropriate inner cell mass (ICM) quality (grade A or B), and acceptable trophectoderm (TE) quality (grade A, B, or C) divided by the number of ICSI oocytes.

### 2.4 Oocyte birefringence detection

We performed PL imaging before ICSI. Oocytes were placed in a glass-bottom dish (Matsunami Glass Ind., Osaka, Japan) in a small (10-μL) drop of HEPES-buffered, prewarmed human tubal fluid medium (Kitazato Corporation, Fuji, Japan) and covered in paraffin oil (Vitrolife, Kungsbacka, Sweden). These oocytes were then placed on a 37 °C heating plate under an inverted microscope. PL images were collected at 400× magnification by using the Oosight Imaging System (CRI, MA, USA). Before imaging, we applied PL filters and calibrated the software for background acquisition. Subsequently, we observed the oocytes in the bright field to adjust the focal plane. The PL images of individual oocytes were then recorded for subsequent analyses. PL parameters, including light retardance values (oocyte and spindle area), spindle size, and angle deviation between the PB1 and MII spindle, were manually measured using Oosight software tools (**Supplementary Figure 1**) by a single senior embryologist to ensure maximal internal consistency for this proof-of-concept study.

### 2.5 Morphokinetic and embryo quality evaluation

At 118 h after insemination, we performed TL assessments of morphokinetics, cleavage dysmorphisms, and morphology for all full-blastocyst embryos by using Embryo Viewer (Vitrolife, Kungsbacka, Sweden)—as outlined in our previous report (28). Time of ICSI was defined as time zero. ICM and TE quality were evaluated according to the manufacturer’s guidelines, in which the Gardner blastocyst grading system is generalized for TL imaging. **Supplementary Table 1** provides details regarding our TL parameters.

### 2.6 Statistical analysis

**Figure 1:**
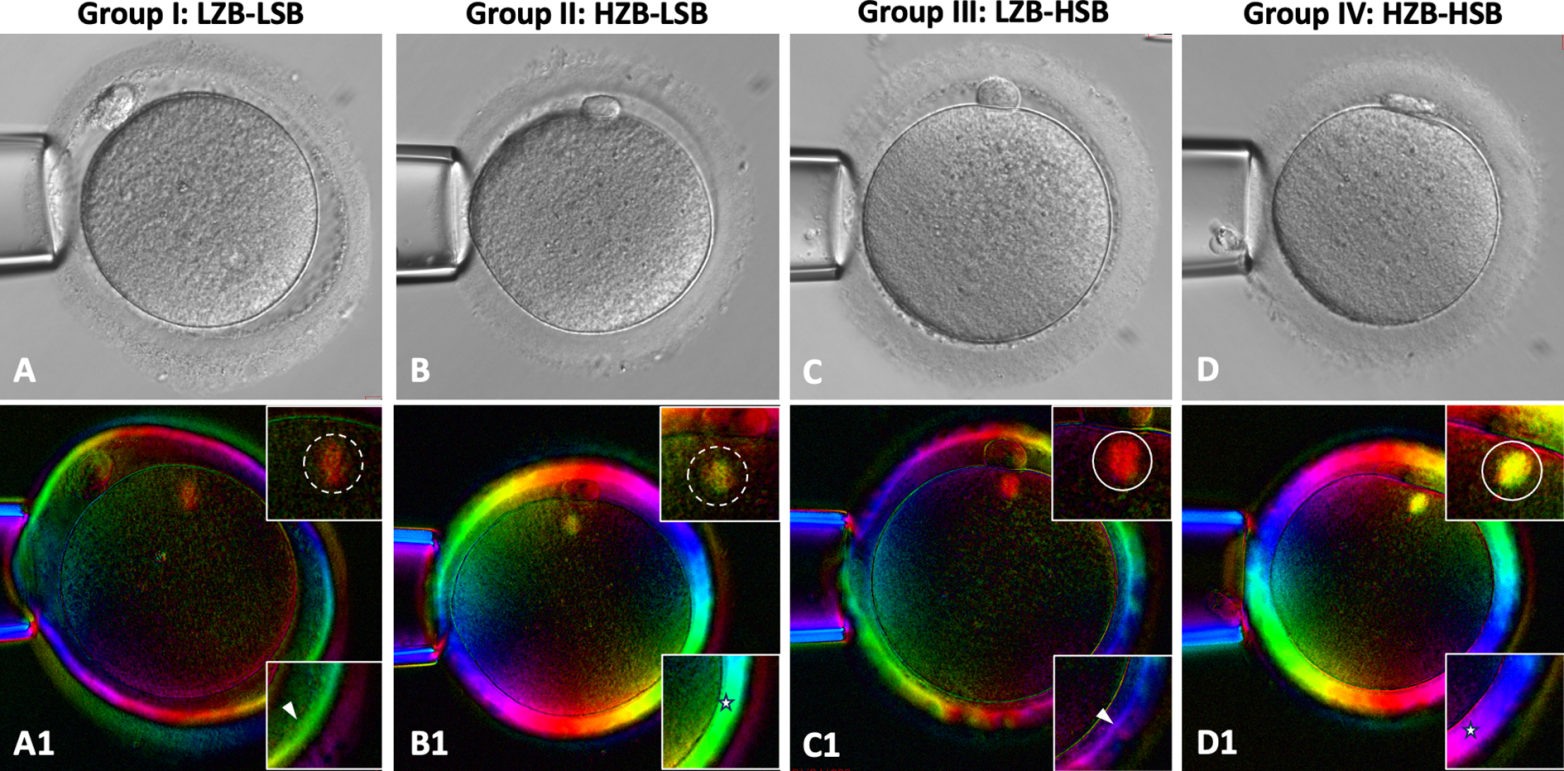
Representative bright-field **(A–D)** and birefringence (A1–D1) images of metaphase II spindle–positive oocytes captured using the Oosight Imaging System. Oocytes were qualitatively classified on the basis of birefringence morphology and signal intensity in both the spindle and zona pellucida (ZP). In the inset figures, dotted circles indicate low spindle birefringence (LSB; characterized by reduced signal intensity), solid circles indicate high spindle birefringence (HSB; characterized by intact spindle morphology), arrowheads denote low ZP birefringence (LZB; characterized by a thin or disrupted inner ZP layer), and stars indicate high ZP birefringence (HZB; characterized by a thick, intact inner ZP layer). Four qualitative groups were identified: (A–A1) Group I (LZB–LSB), (B–B1) Group II (HZB–LSB), (C–C1) Group III (LZB–HSB), and (D–D1) Group IV (HZB–HSB).

We first qualitatively categorized MII oocytes into four groups on the basis of their spindle and ZP birefringence signals (**Figure 1**) and analyzed between-group differences using the Kruskal–Wallis, Pearson’s chi-square, or Fisher’s exact test, as appropriate. To evaluate the reproducibility of quantitative PL parameters, intra-observer agreement was assessed using the intraclass correlation coefficient in a subset of at least 50 oocytes, yielding scores of 0.84–0.96 and demonstrating good consistency. Moreover, analysis of variance or the Cochran–Armitage test was used to determine significant trends between these groups. To identify associations between the parameters and usable blastocyst formation, we used univariate and multivariate logistic regression models within a generalized estimating equation (GEE). The multivariate model was adjusted for confounders identified in the univariate analysis (P < 0.10). We used receiver operating characteristic (ROC) curve analysis to evaluate the performance of predictors of usable blastocyst formation and a paired-sample design to compare the two ROC curves. We maximized Youden’s index (sensitivity + specificity − 1) to determine the SOLRR cut-off value that provided the highest combined discriminative power. The Mann–Whitney U test was employed to assess between-group differences in morphokinetic parameters. All statistical analyses were performed on SPSS (version 26.0; IBM, Armonk, NY, USA), and a P value of <0.05 was considered to indicate significance.

## 3 Results

### 3.1 Patient characteristics and oocyte evaluation

We enrolled 36 IVF patients with an average infertility duration of 2.2 ± 1.9 years. The average age of the male and female participants was 36.8 ± 3.6 and 33.6 ± 3.1 years, respectively. Regarding the baseline clinical parameters, the average AMH level and BMI were 4.6 ± 2.2 ng/mL and 23.3 ± 2.6 kg/m^2^, respectively. On trigger day, the average serum progesterone, estradiol, and LH levels were 1.0 ± 0.6 ng/mL, 5382.4 ± 3531.1 pg/mL, and 3.3 ± 1.9 IU/L, respectively. Through ultrasound-guided ovum pick-up, 21.9 ± 9.3 oocytes were efficiently recovered; of them, an average of 16.1 ± 6.8 oocytes per patient had extruded PB1 and were successfully evaluated using PL microscopy. The PL imaging results demonstrated that 94.5% (549/581) of the PB1-positive oocytes had detectable MII spindle signals (**Supplementary Figure 2**). After ICSI, oocytes with a visible MII spindle demonstrated significantly higher rates of 2PN zygote formation (80.9% vs. 46.9%), full-blastocyst formation (57.9% vs. 18.8%), and usable blastocyst development (46.1% vs. 15.6%) compared with those without. Furthermore, among the 2PN zygotes, oocytes without a detectable MII spindle exhibited a higher prevalence of retained second polar bodies (33.3%), suggesting incomplete progression through meiosis (**Supplementary Table 2**).

### 3.2 PL Parameters and embryonic developmental outcomes

We subsequently assessed oocytes with a visible MII spindle (n = 549; **Figure 1**). We visually categorized oocytes on the basis of their spindle and ZP birefringence signals, defining them as high and low spindle birefringence (HSB and LSB, respectively) and high and low ZP birefringence (HZB and LZB, respectively). Consequently, four distinct subgroups were established (**Table 1**): LZB–LSB (n = 19), HZB–LSB (n = 112), LZB–HSB (n = 81), and HZB–HSB (n = 337).

**Table 1:** The polarized light imaging and embryonic development of MII oocyte groups.

Polarized light imaging and embryo development	LZB-LSB (n = 19)	HZB-LSB (n = 112)	LZB-HSB (n = 81)	HZB-HSB (n = 337)	Trend test
Oocyte light retardance, nm	47376.7 ± 8359.1^ab^	63366.4 ± 13952.3^ac^	47236.8 ± 8092.1^cd^	62696.0 ± 12598.8^bd^	P < 0.05
Spindle light retardance, nm	335.1 ± 109.3^ab^	361.8 ± 152.3^cd^	563.4 ± 143.1^ace^	664.5 ± 172.5^bde^	P < 0.05
Spindle-to-oocyte light retardance ratio	0.007 ± 0.002^ab^	0.006 ± 0.003^cd^	0.012 ± 0.003^ace^	0.011 ± 0.003^bde^	P < 0.05
Spindle size, μm^2^	241.3 ± 83.3^ab^	216.1 ± 70.5^cd^	287.3 ± 40.5^ac^	297.2 ± 73.6^bd^	P < 0.05
Spindle deviation angle, ^∘^	56.6 ± 48.4	33.6 ± 31.1	44.5 ± 30.6	40.4 ± 32.2	NS
Normal fertilization, n (%)	15 (78.9%)	80 (71.4%)^a^	66 (81.5%)	283 (84.0%)^a^	P < 0.05
Full blastocysts, n (%)	7 (36.8%)^a^	49 (43.8%)^cd^	50 (61.7%)^c^	214 (63.5%)^ad^	P < 0.05
Usable blastocysts, n (%)	3 (15.8%)^a^	36 (32.1%)^b^	33 (40.7%)^c^	181 (53.7%)^abc^	P < 0.05
Day 5 blastocysts, n (%)	1 (5.3%)^a^	17 (15.2%)^b^	19 (23.5%)	106 (31.5%)^ab^	P < 0.05
Day 6 blastocysts, n (%)	2 (10.5%)	19 (16.9%)	14 (17.3%)	75 (22.3%)	NS

^a,b,c,d,e^ The same symbols indicate a significant difference in the same column using the Kruskal-Wallis test or Fisher’s exact test. The significant trends between these groups were determined by the Cochran–Armitage test. The abbreviations “HZB”, “LZB”, “HSB”, “LSB”, and “NS” denoted high zona-pellucida birefringence, low zona-pellucida birefringence, high spindle birefringence, low spindle birefringence, and “non-significance”, respectively.

Analysis of PL parameters using Oosight revealed significant differences among the four groups in all birefringence metrics (P < 0.05), with the exception of spindle deviation angle. The HZB–HSB group demonstrated the most favorable oocyte and spindle organization, as indicated by improved oocyte light retardance (OLR; 62696.0 ± 12599.8 nm), spindle light retardance (SLR; 664.5 ± 172.5 nm), SOLRR (0.011 ± 0.003), and spindle size (297.2 ± 73.6 μm^2^). Compared with LSB oocytes, HSB oocytes achieved higher normal fertilization rates (81.5%–84.0% vs. 71.4%–78.9%) and full-blastocyst rates (61.7%–63.5% vs. 36.8%–43.8%). Moreover, HSB–HZB oocytes demonstrated a higher usable blastocyst rate (53.7% vs. 15.8%–40.7%; P < 0.05) and more day 5 blastocysts (31.5% vs. 5.3%–23.5%) than other subgroups did, indicating more favorable blastocyst quality and faster embryonic progression. The Cochran–Armitage trend test further confirmed an upward trend in PL parameters and developmental outcomes, with the exception of spindle deviation angle and proportion of day 6 blastocysts, across subgroups (P < 0.05). Taken together, these findings suggest that high spindle and ZP birefringence are associated with increased fertilization and blastocyst development, most likely due to improved oocyte organization and meiotic spindle integrity (**Table 1**).

### 3.3 Associations between PL parameters and usable blastocyst formation

We evaluated the effects of PL imaging parameters on usable blastocyst formation by using a GEE model (**Table 2**). Univariate analysis revealed that AMH, OLR, SLR, SOLRR, and spindle size were all significantly associated with the likelihood of usable blastocyst formation (P < 0.05). Consequently, AMH, SOLRR, spindle size, and spindle deviation angle were introduced as confounding variables in our multivariate model. In the multivariate analysis, SOLRR was positively associated with usable blastocyst formation (adjusted odds ratio [aOR] = 1.148, 95% confidence interval [CI] = 1.076–1.226; P < 0.001). By contrast, high AMH levels were associated with a reduced probability of this outcome (aOR = 0.939, 95% CI = 0.899–0.982; P = 0.006). Other PL parameters, that is, spindle size and deviation angle, became nonsignificant after adjustment. These findings suggest that ovarian reserve (indicated by AMH) and SOLRR exerted independent effects on the probability of generating usable blastocysts in our cohort.

**Table 2:** Logistic regression analysis was performed to determine the associations between polarized light imaging parameters and usable blastocyst formation.

Parameters	Univariate model				Multivariate model			
	*P* value	OR	95% CI		*P* value	^a^OR	95% CI	
			Lower	Upper			Lower	Upper
Female age, year	0.533	0.978	0.911	1.050	–	–	–	–
Male age, year	0.652	1.012	0.962	1.063	–	–	–	–
BMI, kg/m^2^	0.824	0.992	0.927	1.062	–	–	–	–
AMH, ng/mL	0.002	0.937	0.900	0.976	0.006	0.939	0.899	0.982
Sperm quality: Abnormal vs. Normal^$^	0.584	0.922	0.690	1.232	–	–	–	–
Infertility duration, year	0.327	0.957	0.878	1.044	–	–	–	–
Trigger protocols: Agonist vs. Dual^$^	0.486	0.853	0.545	1.335	–	–	–	–
LH-trigger, IU/L	0.693	1.017	0.934	1.109	–	–	–	–
E2-trigger, pg/mL*	0.628	0.990	0.951	1.031	–	–	–	–
P4-trigger, ng/mL	0.664	0.945	0.733	1.219	–	–	–	–
Oocyte number	0.694	0.997	0.982	1.012	–	–	–	–
Mature oocyte number	0.354	0.991	0.973	1.010	–	–	–	–
Oocyte light retardance*, nm	0.001	0.977	0.963	0.991	–	–	–	–
Spindle light retardance*, nm	0.007	3.930	1.450	10.651	–	–	–	–
Spindle-to-oocyte light retardance ratio^#^	<0.001	1.123	1.068	1.181	<0.001	1.148	1.076	1.226
Spindle size, μm^2^	0.013	1.003	1.001	1.005	0.356	0.999	0.995	1.002
Spindle deviation angle, ^∘^	0.070	1.004	1.000	1.009	0.345	1.002	0.997	1.007

The abbreviations “BMI”, “AMH”, “LH”, “E2”, “P4”, “OR”, “^a^OR”, and “CI” denoted body mass index, anti-Müllerian hormone, luteinizing hormone, estradiol, progesterone, odds ratio, adjusted odds ratio, and confidence interval, respectively. ^$^indicated the reference group. *The odds ratio of usable blastocyst development was calculated per 1000 unit increase. ^#^The odds ratio of usable blastocyst development was calculated per 0.001 unit increase.

### 3.4 Predictive performance of PL parameters for usable blastocyst formation

**Figure 2:**
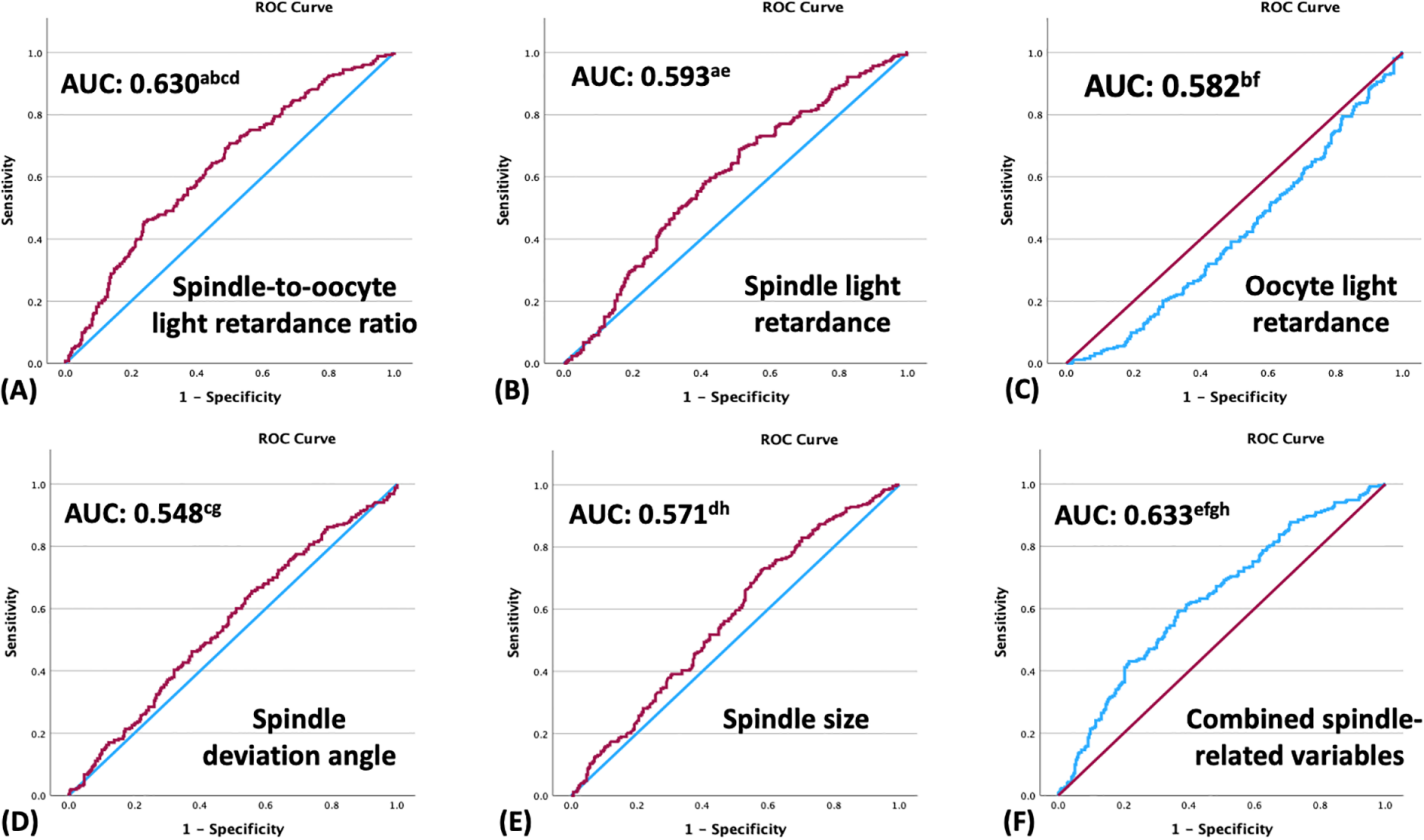
Receiver operating characteristic curves for the prediction of usable blastocyst formation based on polarized light imaging parameters: **(A)** spindle-to-oocyte light retardance ratio (area under the ROC curve [AUC] = 0.630), **(B)** spindle light retardance (AUC = 0.593), **(C)** oocyte light retardance (AUC = 0.582), **(D)** spindle deviation angle (AUC = 0.548), **(E)** spindle size (AUC = 0.571), and **(F)** combined spindle-related variables (spindle-to-oocyte light retardance ratio, spindle area, and spindle deviation angle; AUC = 0.633). Significant differences between AUCs were indicated by identical superscript letters (a,b,c,d,e,f,g,h).

We used ROC curve analysis to assess the predictive ability of the evaluated PL parameters for usable blastocyst formation (**Figure 2**). Of all parameters, the SOLRR demonstrated the strongest predictive ability, yielding an area under the ROC curve (AUC) of 0.630, with a specificity of 76.0%, a sensitivity of 45.5%, and a precision of 61.8%. By contrast, the SLR (AUC = 0.593), the OLR (AUC = 0.582), the spindle deviation angle (AUC = 0.548), and spindle size (AUC = 0.571) demonstrated relatively low predictive ability (P < 0.05). When the SOLRR was combined with spindle deviation angle and spindle size, the AUC only marginally increased to 0.633, which is comparable to that of the SOLRR alone.

**Figure 3:**
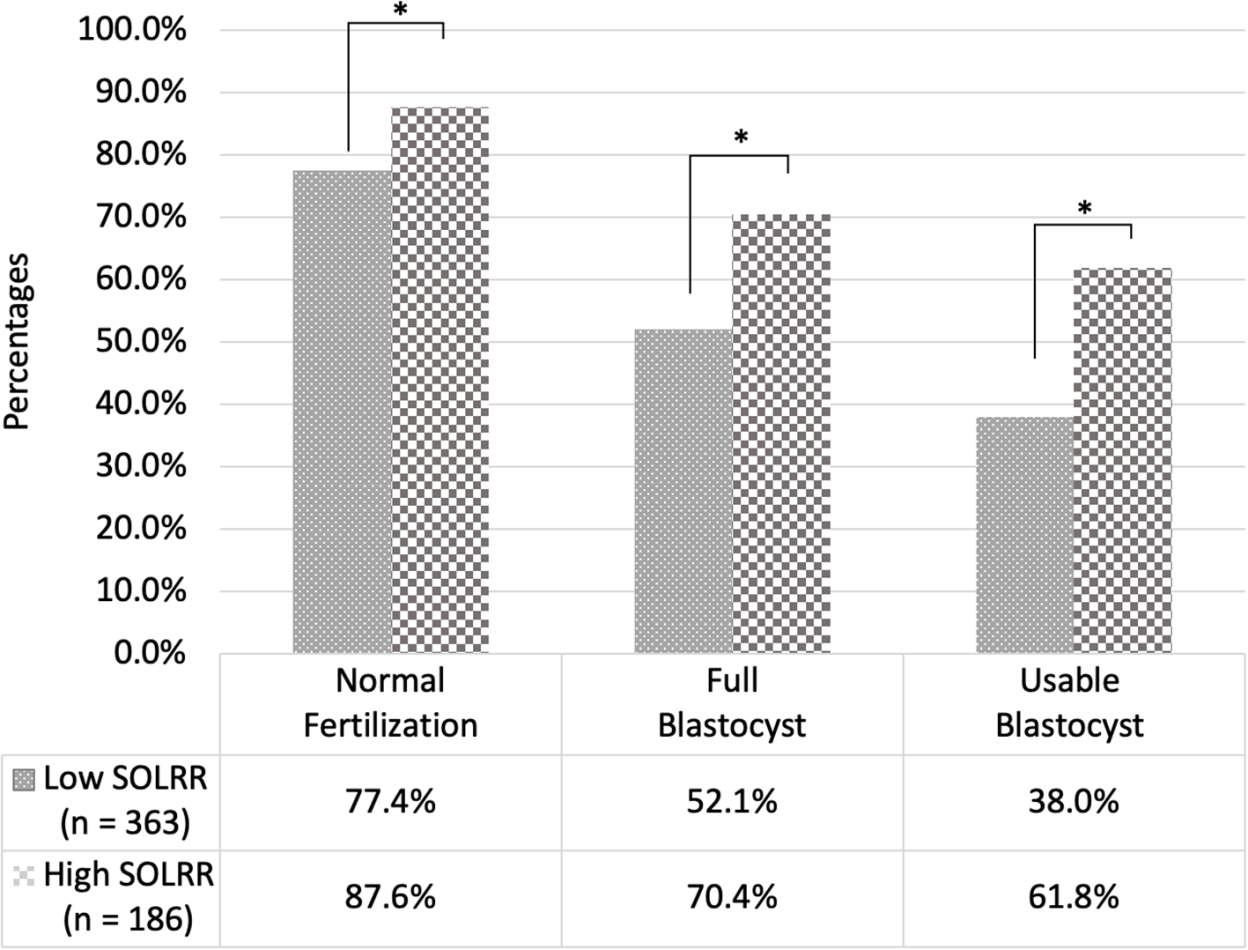
Comparison of normal fertilization, full-blastocyst development, and usable blastocyst formation rates between high (≥0.01127) and low (<0.01127) spindle-to-oocyte light retardance ratio (SOLRR) groups. The high SOLRR group exhibited significantly higher rates (87.6%, 70.4%, and 61.8%, respectively) compared with the low SOLRR group (77.4%, 52.1%, and 38.0%, respectively), indicating the predictive value of the SOLRR for identifying competent oocytes.

Based on the maximization of Youden’s index, an optimal SOLRR cutoff value of 0.01127 was derived. Oocytes were subsequently categorized into high (≥0.01127) or low (<0.01127) SOLRR groups. The high SOLRR group consistently demonstrated significantly higher rates of normal fertilization (87.6% vs. 77.4%), full-blastocyst development (70.4% vs. 52.1%), and usable blastocyst formation (61.8% vs. 38.0%) relative to the low SOLRR group (**Figure 3**). After adjusting for intra-patient clustering using GEE models, the high SOLRR group consistently maintained a significantly greater probability of reaching key developmental endpoints, including usable blastocyst formation (**Supplementary Table 3**). Thus, SOLRR may have a moderate ability to predict an oocyte’s potency to efficiently develop into a usable blastocyst.

### 3.5 Embryonic morphokinetics, dysmorphisms, and morphology between SOLRR groups

TL assessments were used to analyze embryonic morphokinetics, dysmorphisms, and morphology in embryos that reached the full-blastocyst stage (n = 320). The median time of developmental progression to the blastocyst stage was significantly shorter in the high SOLRR group than in the low SOLRR group across various timepoints: tPB2 (1.7 vs. 2.02 h; P = 0.016), tPNf (21.8 vs. 22.3 h; P = 0.015), t2 (24.6 vs. 25.1 h; P = 0.006), t3 (35.7 vs. 36.5 h; P = 0.018), t4 (36.3 vs. 37.3 h; P = 0.003), t5 (48.9 vs. 50.8 h; P = 0.006), t8 (53.9 vs. 57.1 h; P = 0.001), tM (88.3 vs. 91.3 h; P = 0.026), and tB (108.2 vs. 111.2 h; P = 0.008). However, the between-group differences in time intervals during early cleavage stages (i.e., CC2, CC3, S2, and S3) were not significant. Nevertheless, the interval from tSB to tB was significantly shorter in the high SOLRR group than in the low SOLRR group (8.7 vs. 10.2 h; P = 0.033; **Table 3**).

**Table 3:** Morphokinetic analysis for the full-blastocyst embryos derived from MII oocytes.

*Morphokinetics	^#^Low spindle-to-oocyte light retardance ratio (n = 189)		^$^High spindle-to-oocyte light retardance ratio (n = 131)		*P* value
	Median	25^th^-75^th^ percentiles	Median	25^th^-75^th^ percentiles	
tPB2, h	2.02	1.4–2.7	1.7	1.4–2.2	0.016
tPNf, h	22.3	20.5–24.6	21.8	19.6–23.5	0.015
t2, h	25.1	23.1–27.3	24.6	22.4–26.1	0.006
t3, h	36.5	34.0–39.0	35.7	33.0–38.1	0.018
t4, h	37.3	35.0–40.9	36.3	33.8–38.8	0.003
t5, h	50.8	46.9–54.6	48.9	45.3–52.6	0.006
t8, h	57.1	51.8–69.8	53.9	49.7–59.8	0.001
tM, h	91.3	84.9–96.7	88.3	83.6–95.1	0.026
tSB, h	100.5	94.6–106.8	98.9	93.9–104.5	0.100
tB, h	111.2	104.6–120.1	108.2	101.8–115.4	0.008
CC2 (t3-t2), h	11.4	10.7–12.2	11.2	10.4–12.2	0.194
S2 (t4-t3), h	0.5	0.2–1.4	0.5	0.2–1.0	0.552
CC3 (t5-t3), h	14.0	12.6–15.4	13.7	12.2–15.2	0.211
S3 (t8-t5), h	4.9	2.6–15.1	4.0	2.4–8.9	0.084
t5-t2, h	25.5	23.6–27.5	24.9	22.8–26.9	0.102
tSB-tB, h	10.2	7.4–13.3	8.7	6.8–12.1	0.033

The Mann-Whitney U test was used to assess differences between groups. *Time of ICSI was defined as time zero (t0). Time-lapse parameters were defined in **Supplementary Table 1**. ^#^Spindle-to-oocyte light retardance ratio < 0.01127. ^$^Spindle-to-oocyte light retardance ratio ≥ 0.01127.

Most embryonic dysmorphisms and morphological characteristics, including pronuclear morphology, synchronized pronuclear fading, cleavage patterns (i.e., Even2 and Even4), multinucleation (i.e., MN2 and MN4), and division abnormalities (i.e., incomplete chaotic division and direct uneven cleavage) were comparable between the groups (**Table 4**). Nevertheless, the high SOLRR group demonstrated a higher nonreverse cleavage rate (95.4% vs. 86.8%; P = 0.012) and more favorable TE morphology on day 5 (i.e., TE ≥ B grades, 58.0% vs. 40.7%; P = 0.003) than the low SOLRR group did. Finally, the fragmentation rate at the 8-cell stage, cytoplasmic vacuoles, and ICM grade did not differ significantly between the groups. Moreover, adjusting for within-patient clustering using GEE models confirmed our primary findings. Embryos in the high SOLRR cohort maintained significantly better TE morphology and faster cleavage kinetics at key developmental checkpoints (**Supplementary Table 3**).

## 4 Discussion

Oocyte competence directly influences fertilization, embryonic development, and reproductive outcomes; thus, its assessment remains a primary objective in contemporary IVF protocols (1). Although traditional morphological grading is the clinical standard, it is subject to bias and fails to reflect intrinsic cytoplasmic and chromosomal quality, indicating a need for improved tools (29, 30). PL imaging is an attractive noninvasive alternative to conventional methods for oocyte quality assessment. In this technique, oocyte structural organization can be assessed in real time and integrated into the IVF workflow seamlessly, without compromising viability (5, 22). Studies have established the prognostic value of qualitative spindle evaluation, reporting that a visible MII spindle is correlated with enhanced fertilization and improved embryo development (8–10); consequently, meiotic spindle integrity is considered a hallmark of oocyte maturity and the appropriate timing for insemination (31, 32). The current results confirm the importance of visualizing the MII spindle before ICSI. Oocytes with a visible MII spindle demonstrated significantly improved potential for both fertilization and subsequent blastocyst development (**Supplementary Table 1**). Notably, TL assessments revealed an increased prevalence of abnormal 2PN formation, characterized by incomplete meiosis and failure to extrude PB2 after ICSI in anaphase I or telophase I oocytes.

In addition to MII spindle visualization, ZP birefringence has been proposed as a marker of oocyte competence. HZB may be associated with favorable outcomes of embryonic development and pregnancy (5). Nevertheless, the importance of both MII spindle visibility and ZP characteristics has complicated their clinical application of either parameter because which of these optical parameters more reliably reflects intrinsic oocyte quality remains uncertain. To our knowledge, our study is the first to simultaneously categorize oocytes into four distinct groups on the basis of MII spindle and ZP birefringence signals (**Figure 1**). This integrative approach demonstrated developmental stratification, underscoring the synergistic effects of combining the aforementioned signals for effective oocyte selection. The current results confirm an upward trend in normal fertilization and blastocyst development rates across the groups, with the HZB–HSB group achieving the most favorable outcomes (**Table 1**).

To ensure analytical rigor and consistency, we validated these qualitative birefringence classifications by using Oosight. The results confirm that the birefringence-defined subgroups exhibited corresponding differences in quantitative SLR and OLR (**Table 1**). Moreover, research reported that MII spindle geometry is correlated with developmental potential (33). High-quality human MII oocytes, identified by early cleavage, exhibit larger spindles than lower-quality oocytes do (34). In particular, achieving an optimal MII spindle size is correlated with an improved clinical pregnancy rate (35). In the current study, in LSB oocytes, the spindle size was relatively small (**Table 1**), indicating that they were likely less mature MII oocytes (32). Notably, spindle size was positively associated with usable blastocyst formation (odds ratio [OR] = 1.003, P < 0.05; **Table 2**). By contrast, the spindle deviation angle neither differed among subgroups nor was correlated with usable blastocyst development (**Tables 1**, **2**), corroborating previous findings by indicating that deviation relative to the PB1 may reflect procedural artifacts rather than intrinsic oocyte biology (20, 36).

In the current study, the results of qualitative birefringence assessments performed by an experienced embryologist were consistent; however, this qualitative interpretation was inherently operator-dependent. Thus, we explored quantitative birefringence metrics for a relatively objective assessment. Notably, the OLR demonstrated an inverse association with usable blastocyst formation (OR = 0.977, P < 0.05), whereas the SLR exhibited a positive association (OR = 3.930, P < 0.05) (**Table 2**). Studies have suggested that the OLR primarily reflects the characteristics of the inner ZP layer (**Figure 1**), thereby indicating overall structural integrity and optimal cytoplasmic potential. Oocytes with high birefringence might have the optimal competence for embryonic growth and implantation (5, 11). However, herein, we observed a contrasting result, suggesting that OLR might be influenced by MII spindle formation (i.e., SLR) or by other confounding factors. This discrepancy may be partially explained by the high-responder nature of our cohort. The patients were generally young (33.6 ± 3.1 years) with high ovarian reserve (AMH 4.6 ± 2.2 ng/mL), yielding large oocyte cohorts (21.9 ± 9.3 oocytes). Robust follicular recruitment frequently induces maturation asynchrony (37), and premature fertilization of late-maturing oocytes can result in poor embryonic development (32). Therefore, even oocytes presenting with HZB might still harbor underlying nuclear or cytoplasmic immaturity, which ultimately impairs subsequent embryonic development. Consequently, our results clarified the previously inconclusive results for clinical application of quantitative birefringence (3, 20, 22, 23), underscoring the limitations of relying on OLR or SLR alone.

The SOLRR, for which oocyte and spindle birefringence are considered simultaneously, emerged as an exploratory, biologically-meaningful quantitative marker in the present study. The SOLRR was elevated in HSB subgroups (i.e., LZB–HSB and HZB–HSB), indicating an increased potential for normal fertilization and blastocyst formation (**Table 1**). In addition, the SOLRR demonstrated significant associations with usable blastocyst development in both unadjusted (OR = 1.123) and adjusted (aOR = 1.148) analyses (**Table 2**). The evaluation of the predictive ability of quantitative PL parameters for usable blastocyst formation (**Figure 2**) strongly supported our concept. The SOLRR (AUC = 0.630) significantly outperformed other individual parameters, including the SLR, the OLR, spindle deviation angle, and spindle size (AUCs = 0.548–0.593). These data highlighted that OLR provided a necessary physiological denominator. By contextualizing spindle intensity relative to its immediate cytoplasmic and ZP background, the SOLRR mitigates the confounding effects of absolute brightness variations among individual oocytes, offering stronger predictive power than isolated retardance metrics. Normal fertilization and blastocyst development rates were higher in the high SOLRR group than in the low SOLRR group (**Figure 3**). Taken together, these results suggest that the SOLRR can overcome the previously noted inconsistencies in PL-based oocyte assessment and thus represents an exploratory, noninvasive, and quantitative biomarker for the developmental competence of oocytes. However, it is also noticed that SOLRR has a moderate predictive value and low sensitivity. As such, SOLRR should be used in conjunction with standard morphological and morphokinetic evaluations to provide complementary biophysical insights, rather than as a stand-alone metric in the current condition.

Several studies have demonstrated that faster morphokinetic progression consistently correlates with higher embryo competence, implantation potential, and euploidy rates, establishing TL monitoring as an effective adjunctive tool for embryo selection (28, 38–41). In the present study, embryos derived from high SOLRR oocytes exhibited more synchronized, timely development than did those from low SOLRR oocytes (**Table 3**). The low SOLRR group demonstrated delayed initiation at the tPB2, strongly suggesting incomplete cytoplasmic maturation or spindle dysfunction; it also exhibited increased abnormal cleavage risk and impaired embryonic developmental potential (42, 43). TL analysis results consistently demonstrated that delayed PB2 extrusion predicted slower subsequent timings (e.g., tPNf and early cleavages) and increased the risks of abnormal cleavage or failure to achieve a high-quality blastocyst (42, 44). We also discovered that developmental delays in low SOLRR oocytes persisted in the subsequent cleavage events (i.e., tPNf, t2, t3, t4, and t5) and were linked to suboptimal embryo development (**Table 3**, **Figure 3**). In addition, we observed more pronounced delays at t8, a critical point coinciding with human embryonic genome activation. Perturbations at this transition may disrupt zygotic transcriptional initiation and reduce developmental potential (43, 45, 46). Moreover, delayed compaction (tM) and prolonged blastulation (tSB–tB), which were noted in the low SOLRR group, may be associated with altered gene expression critical for lineage specification and blastocoel formation (47, 48), ultimately resulting in reduced blastocyst quality (**Table 4**) and pregnancy or obstetric outcomes (49–51). These results also indicate an increased incidence of reverse cleavage in full blastocysts derived from low SOLRR oocytes (**Table 4**), which correlated directly with poor pregnancy outcomes (52). These findings collectively indicate that early kinetic delays and dysmorphisms observed in low SOLRR oocytes potentially originated from suboptimal oocyte maturity, propagated throughout preimplantation development, and culminated in impaired blastocyst formation and reduced embryo competence.

**Table 4:** Embryonic dysmorphisms and morphology for the full-blastocyst embryos derived from MII oocytes.

Embryonic dysmorphisms and morphology	^#^Low spindle-to-oocyte light retardance ratio (n = 189)	^$^High spindle-to-oocyte light retardance ratio (n = 131)	*P* value
Even PN size, % (n)	68.8% (130)	59.5% (78)	0.096
Synchronized PN fading, % (n)	98.9% (187)	99.2% (130)	1.000
Even2, % (n)	89.9% (170)	93.9% (123)	0.228
Even4, % (n)	75.7% (143)	80.2% (105)	0.414
Non-MN2, % (n)	71.4% (135)	74.0% (97)	0.703
Non-MN4, % (n)	84.1% (159)	86.3% (113)	0.636
Non-ICD, % (n)	94.2% (178)	96.2% (126)	0.603
Non-DUC, % (n)	94.2% (178)	92.4% (121)	0.647
Non-RC, % (n)	86.8% (164)	95.4% (125)	0.012
Non-vacuoles, % (n)	78.8% (149)	79.4% (104)	1.000
Fragment8 < 25%, % (n)	93.1% (176)	95.4% (125)	0.476
ICM ≥ B grades, % (n)	67.7% (128)	76.3% (100)	0.103
TE ≥ B grades, % (n)	40.7% (77)	58.0% (76)	0.003

The Fisher’s exact test was used to assess differences between groups. Time-lapse parameters were defined in **Supplementary Table 1**. ^#^Spindle-to-oocyte light retardance ratio < 0.01127. ^$^Spindle-to-oocyte light retardance ratio ≥ 0.01127.

This study has several limitations. First, because this was an observational cohort study, we could not establish definitive causality between oocyte birefringence characteristics and developmental outcomes. Although valuable for hypothesis generation, the current study design is susceptible to selection bias, unmeasured confounding, and reverse causation. Nevertheless, our results demonstrate that confounding factors, such as baseline ovarian reserve (i.e., AMH), also affect oocyte quality. High AMH levels, often associated with polycystic ovary syndrome (53), can alter spindle characteristics and cytoplasmic competence (37); in the current study, they were negatively associated with usable blastocyst formation (**Table 2**). Future randomized controlled trials incorporating SOLRR-guided oocyte selection are warranted to definitively confirm the causal effects of PL parameters on blastocyst formation or subsequent pregnancy outcomes, as these clinical outcomes were not assessed in this study. Second, the generalizability of our findings is limited by our inclusion criteria, which focused on relatively young women (≤ 38 years) with favorable ovarian reserves (AMH > 1.1 ng/mL). Consequently, our results may not directly translate to patients with advanced maternal age or poor ovarian reserve, whose oocytes may exhibit different spindle and ZP birefringence characteristics due to age-related oxidative stress or mitochondrial dysfunction (1, 2). Future studies evaluating the utility of the SOLRR in these compromised populations are essential to determine its broader clinical applicability and to establish whether the proposed threshold remains consistent across different patient demographics. Finally, the requirement for specialized equipment and additional analysis time may constrain the routine use of SOLRR in high-throughput IVF laboratories.

## 5 Conclusions

Birefringence quantification based on PL imaging provides a noninvasive, objective assessment of oocyte competence. Herein, we noted that oocytes with strong MII spindle and ZP birefringence have high developmental potential. The SOLRR emerged as an exploratory quantitative indicator, independently predicting higher usable blastocyst formation, faster embryonic development, and superior trophectoderm morphology in oocytes. Because our current dataset does not include clinical pregnancy outcomes, we propose using SOLRR as an adjunctive measure of oocyte potential. Integrating this ratio into standard IVF selection protocols will first require prospective validation with definitive clinical endpoints.

## Data Availability

The original contributions presented in the study are included in the article/**Supplementary Material**. Further inquiries can be directed to the corresponding authors.

## Supplementary material

Supplementary Figure 1 Illustration of the manual measurement methodology for polarized light parameters. **(A)** Schematic representation of the four-step mechanism (generated with the assistance of Google NotebookLM): (1) Entry of polarized light through a linear polarizer; (2) Interaction with highly ordered biological structures (ZP glycoproteins and spindle microtubules); (3) Splitting of light into fast (ordinary) and slow (extraordinary) rays; and (4) Formation of a phase shift, where the retardation (measured in nm) creates visible contrast for clinical assessment. **(B)** Bright-field image and **(C)** corresponding birefringence image of an MII oocyte captured using the Oosight Imaging System. In the birefringence image **(C)**, the large red dashed circle indicated the boundary used to measure oocyte light retardance. The small white dashed ellipse defined the specific area used for measuring spindle light retardance and spindle size. The blue lines illustrated the calculation of the spindle deviation angle relative to polar body 1.

Supplementary Figure 2 Representative birefringence images of polar body 1 (PB1)-positive oocytes captured using Oosight. Oocytes with the PB1 were qualitatively classified on the basis of spindle birefringence morphology and signal intensity, including **(A)** anaphase I or telophase I oocyte with a strong, long spindle; **(B)** telophase I oocyte with a weak, long spindle; **(C)** prometaphase II oocyte with no detectable spindle; **(D)** metaphase II oocyte with an extremely weak spindle; **(E)** metaphase II oocyte with a weak spindle; and **(F)** metaphase II oocyte with an intact spindle.
