# Supplementary material for: Spindle-to-oocyte light retardance ratio as a noninvasive biomarker for oocyte quality assessment: a prospective cohort study
Source: Front Endocrinol (Lausanne). 2026 May 5;17:1803476. doi: 10.3389/fendo.2026.1803476 (PMC13183520; doi:10.3389/fendo.2026.1803476)
Supplement: Supplementary file 1 [file Table1.docx]

Supplementary Table 1. The definition of morphokinetics and morphological dysmorphisms.

| Parameters | Definitions |
| --- | --- |
| t0 | time for intracytoplasmic sperm injection |
| tPB2 | time for PB2 extrusion |
| tPNf | time for both pronuclei fading |
| tN | time for embryo accomplishing divisions to N cells |
| tM | time for embryo accomplishing compaction |
| tSB | time for embryo starting blastocoel formation |
| tB | time for blastocoel cavity starting to push zona pellucida |
| CC2 | the time period of the second cell cycle (t2-t3) |
| S2 | the time period of synchrony of the second cell cycle (t3-t4) |
| CC3 | the time period of the third cell cycle (t3-t5) |
| S3 | the time period of synchrony of the third cell cycle (t5-t8) |
| t2-t5 | the time period between t2 and t5 |
| tSB-tSB | the time period between tSB and tB (tSB-tB) |
| Even PN size | the area ratio between two pronuclei immediately before pronuclear breakdown ≥ 0.9 |
| Synchronized PN fading | simultaneous breakdown of two pronuclei |
| Even2 | the area ratio between two blastomeres < 50% at the 2-cell stage |
| Even4 | the area ratio between two blastomeres < 50% at the 4-cell stage |
| Non-MN2 | no multinucleation (single or complex) within individual blastomeres at the 2-cell stage |
| Non-MN4 | no multinucleation (single or complex) within individual blastomeres at the ~~4~~-cell stage |
| Non-ICD | no signs of struggling division (e.g., irregular blebbing, membrane ruffling, or pseudo-furrows), resulting in massive fragmentation (≥ 25%) during the first division |
| Non-DC | no single blastomere divided directly or rapidly (< 5 hours) from 1 cell to 3 cells at the first, second, or third cleavage stage |
| Non-RC | no abnormal cell refusion at the first, second, or third cleavage |
| Non-vacuoles | no de novo formation of vacuoles during in vitro development |
| Fragment8 < 25% | the fragmentation ratio < 25% at the 8-cell stage |
| ICM ≥ B grades | inner cell mass grading ≥ B around 118 hours after ICSI |
| TE ≥ B grades | trophectoderm grading ≥ B around 118 hours after ICSI |
