# Supplementary material for: Spindle-to-oocyte light retardance ratio as a noninvasive biomarker for oocyte quality assessment: a prospective cohort study
Source: Front Endocrinol (Lausanne). 2026 May 5;17:1803476. doi: 10.3389/fendo.2026.1803476 (PMC13183520; doi:10.3389/fendo.2026.1803476)
Supplement: Supplementary file 2 [file Table2.docx]

Supplementary Table 2. Comparison of embryonic *in vitro* development of oocytes with and without MII spindles identified using Oosight.

| Groups | Number | Two-pronuclei zygote formation | *No polar body 2  extrusion | Full-blastocyst formation | Usable blastocyst development |
| --- | --- | --- | --- | --- | --- |
| with metaphase II spindle | 549 | 444 (80.9%)^abc^ | 0 (0%)^ab^ | 318 (57.9%)^abc^ | 253 (46.1%)^abc^ |
| without metaphase II spindle | 32 | 15 (46.9%)^a^ | 5 (33.3%)^a^ | 6 (18.8%)^a^ | 5 (15.6%)^a^ |
| Anaphase-Telophase I | 17 | 10 (58.8%)^b^ | 5 (50.0%)^b^ | 4 (23.5%)^b^ | 3 (17.6%)^b^ |
| Not detected | 15 | 5 (33.3%)^c^ | 0 (0%) | 2 (13.3%)^c^ | 2 (13.3%)^c^ |

^a, b, c^ The same symbols indicate a significant difference in the same column using Fisher’s exact test. *The rate was calculated by dividing the number of two-pronuclei zygotes that failed to extrude a second polar body by the total number of two-pronuclei zygotes.
