# Supplementary material for: Spindle-to-oocyte light retardance ratio as a noninvasive biomarker for oocyte quality assessment: a prospective cohort study
Source: Front Endocrinol (Lausanne). 2026 May 5;17:1803476. doi: 10.3389/fendo.2026.1803476 (PMC13183520; doi:10.3389/fendo.2026.1803476)
Supplement: Supplementary file 3 [file Table3.docx]

Supplementary Table 3. Generalized estimating equation analysis of embryonic kinetics, morphology, and developmental outcomes in high (≥ 0.01127) vs. low (< 0.01127) SOLRR groups^#^.

| Parameters | *P* value | OR | 95% CI | |
| --- | --- | --- | --- | --- |
|  |  |  | Lower | Upper |
| **Developmental outcomes** | | | | |
| Normal fertilization | < 0.05 | 2.165 | 1.214 | 3.86 |
| Full blastocyst formation | < 0.05 | 2.332 | 1.694 | 3.209 |
| Usable blastocyst formation | < 0.05 | 2.639 | 1.96 | 3.551 |
| **Kinetic outcomes**^*^ | | | | |
| tPB2 | < 0.05 | 0.723 | 0.566 | 0.925 |
| tPNf | 0.059 | 0.402 | 0.156 | 1.034 |
| t2 | < 0.05 | 0.337 | 0.127 | 0.891 |
| t3 | 0.090 | 0.317 | 0.084 | 1.199 |
| t4 | < 0.05 | 0.167 | 0.044 | 0.636 |
| t5 | < 0.05 | 0.159 | 0.028 | 0.894 |
| t8 | < 0.05 | 0.011 | 0.001 | 0.153 |
| tM | 0.121 | 0.106 | 0.006 | 1.805 |
| tSB | 0.183 | 0.138 | 0.007 | 2.54 |
| tB | < 0.05 | 0.04 | 0.002 | 0.761 |
| CC2 (t3-t2) | 0.811 | 0.938 | 0.556 | 1.583 |
| S2 (t4-t3) | < 0.05 | 0.549 | 0.342 | 0.882 |
| CC3 (t5-t3) | 0.104 | 0.525 | 0.241 | 1.143 |
| S3 (t8-t5) | < 0.05 | 0.088 | 0.013 | 0.583 |
| t5-t2 | 0.168 | 0.491 | 0.179 | 1.351 |
| tSB-tB | < 0.05 | 0.281 | 0.079 | 0.999 |
| **Morphological outcomes^*^** | | | | |
| Even PN size | 0.140 | 0.664 | 0.386 | 1.143 |
| Synchronized PN fading | 0.631 | 1.391 | 0.361 | 5.356 |
| Even2 | 0.180 | 1.712 | 0.781 | 3.753 |
| Even4 | 0.261 | 1.302 | 0.822 | 2.063 |
| Non-MN2 | 0.372 | 1.26 | 0.759 | 2.091 |
| Non-MN4 | 0.676 | 1.14 | 0.617 | 2.108 |
| Non-ICD | 0.369 | 1.575 | 0.585 | 4.245 |
| Non-DUC | 0.442 | 0.732 | 0.33 | 1.622 |
| Non-RC | 0.062 | 3.193 | 0.942 | 10.825 |
| Non-vacuoles | 0.780 | 1.082 | 0.625 | 1.872 |
| Fragment8 < 25% | 0.338 | 1.536 | 0.639 | 3.692 |
| ICM ≥ B grades | 0.140 | 1.54 | 0.868 | 2.732 |
| TE ≥ B grades | < 0.05 | 2.031 | 1.259 | 3.277 |

The abbreviations “OR” and “CI” denoted odds ratio and confidence interval, respectively. ^*^Time-lapse parameters were defined in Supplementary Table 1. ^#^The group with the lowest spindle-to-oocyte light retardance ratio served as the reference.
