# Supplementary figures and images for: Spindle-to-oocyte light retardance ratio as a noninvasive biomarker for oocyte quality assessment: a prospective cohort study
Source: Front Endocrinol (Lausanne). 2026 May 5;17:1803476. doi: 10.3389/fendo.2026.1803476 (PMC13183520; doi:10.3389/fendo.2026.1803476)

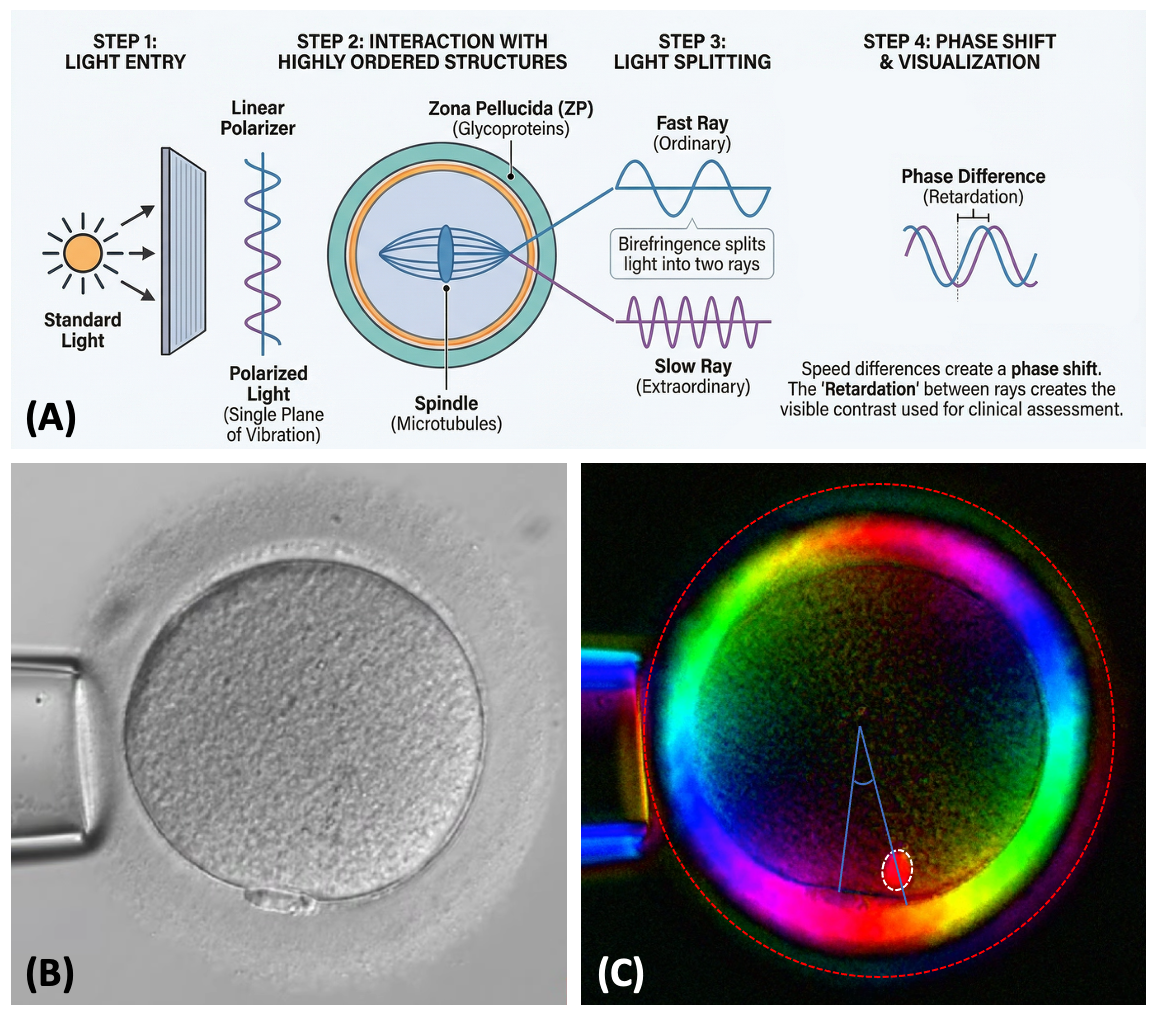

Supplement: Supplementary Figure 1 — Illustration of the manual measurement methodology for polarized light parameters. (A) Schematic representation of the four-step mechanism (generated with the assistance of Google NotebookLM): (1) Entry of polarized light through a linear polarizer; (2) Interaction with highly ordered biological structures (ZP glycoproteins and spindle microtubules); (3) Splitting of light into fast (ordinary) and slow (extraordinary) rays; and (4) Formation of a phase shift, where the retardation (measured in nm) creates visible contrast for clinical assessment. (B) Bright-field image and (C) corresponding birefringence image of an MII oocyte captured using the Oosight Imaging System. In the birefringence image (C), the large red dashed circle indicated the boundary used to measure oocyte light retardance. The small white dashed ellipse defined the specific area used for measuring spindle light retardance and spindle size. The blue lines illustrated the calculation of the spindle deviation angle relative to polar body 1. [file Image1.tiff]

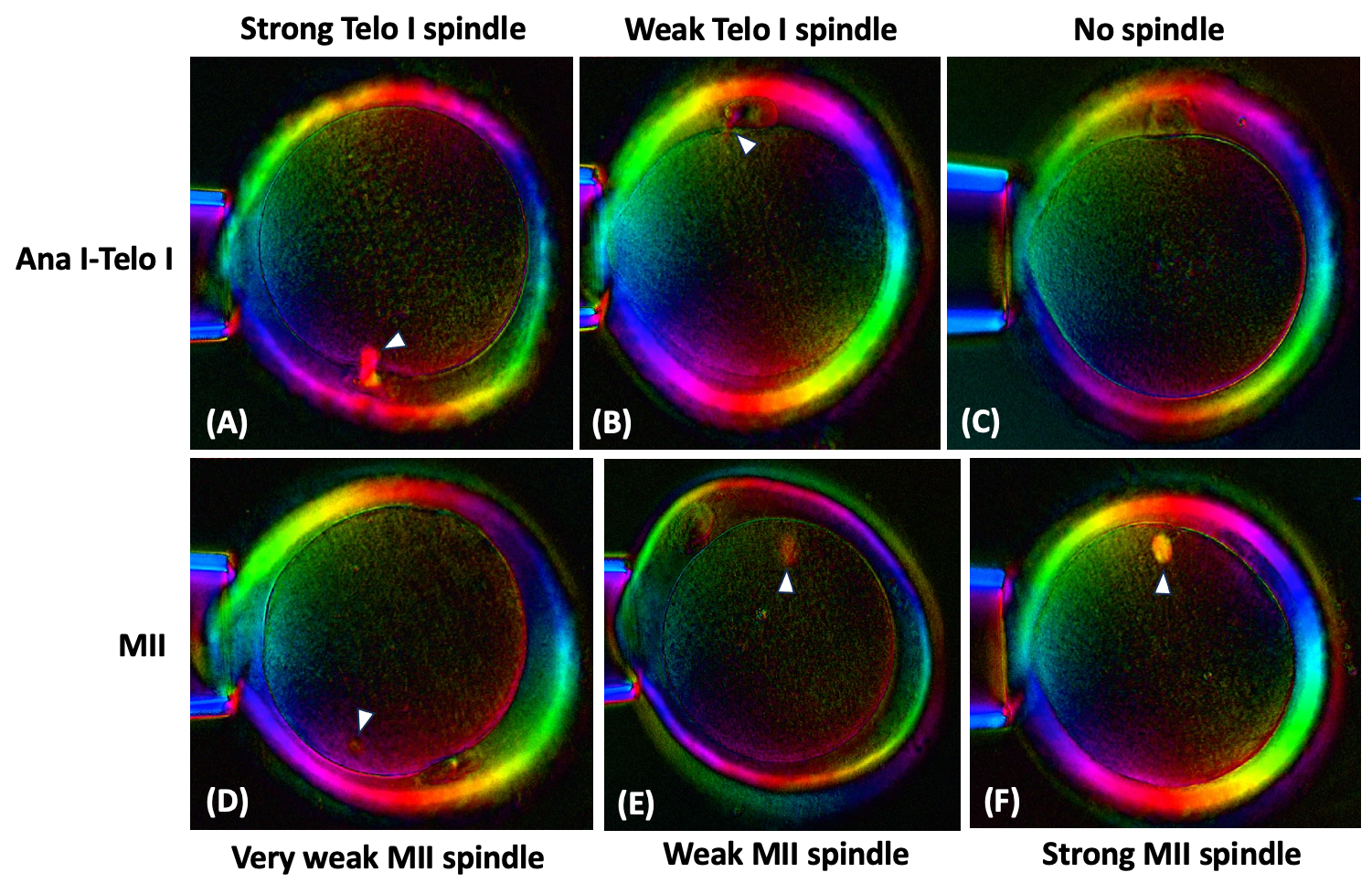

Supplement: Supplementary Figure 2 — Representative birefringence images of polar body 1 (PB1)-positive oocytes captured using Oosight. Oocytes with the PB1 were qualitatively classified on the basis of spindle birefringence morphology and signal intensity, including (A) anaphase I or telophase I oocyte with a strong, long spindle; (B) telophase I oocyte with a weak, long spindle; (C) prometaphase II oocyte with no detectable spindle; (D) metaphase II oocyte with an extremely weak spindle; (E) metaphase II oocyte with a weak spindle; and (F) metaphase II oocyte with an intact spindle. [file Image2.tiff]
